# Transorbital Penetrating Intracranial Injury by Wooden Foreign Body: A Rare Case Report

**DOI:** 10.1002/ccr3.71347

**Published:** 2025-10-27

**Authors:** Uttam Chaulagain, Kapil Khanal, Kailash Mani Pokhrel, Nischal Soti, Urusha Naaz, Prabhat Jha, Dipendra K. Shrestha, Sushil Kumar Shilpakar

**Affiliations:** ^1^ Department of General Surgery Tribhuvan University Teaching Hospital Kathmandu Nepal; ^2^ Institute of Medicine Tribhuvan University, Maharajgunj Medical Campus Kathmandu Nepal; ^3^ Department of Neurosurgery Tribhuvan University Teaching Hospital Kathmandu Nepal

**Keywords:** ACA injury, case report, orbital trauma, transorbital penetrating intracranial injury, wooden foreign body

## Abstract

Transorbital penetrating intracranial injury (TOPI) is a rare form of traumatic brain injury, often caused by high‐velocity projectiles. However, non‐missile penetrating injuries, particularly from wooden foreign bodies, are uncommon and pose diagnostic and therapeutic challenges due to their radiolucency and high risk of infection. We report the case of a 58‐year‐old Nepali male who sustained a TOPI after falling from a tree, with a wooden foreign body penetrating his right orbit and extending intracranially. The patient presented 6 days post‐injury with persistent pain and bleeding but no neurological deficits. Imaging revealed a comminuted fracture of the superomedial orbital wall with intracranial extension, an intraorbital foreign body, and vascular compromise in the anterior cerebral artery (ACA) territory. Given the intracranial extension and vascular involvement, a multidisciplinary approach involving neurosurgery and ophthalmology was employed. The foreign body was successfully removed via a medial orbitotomy, and the patient was managed with broad‐spectrum antibiotics. His postoperative course was uneventful, with no long‐term complications. TOPI involving wooden foreign bodies presents diagnostic difficulties due to their variable CT attenuation and potential to mimic pneumocephalus. MRI is often required for definitive diagnosis, but in resource‐limited settings, a combination of clinical evaluation and CT with angiography can guide management. Early surgical removal and infection control are critical for a favorable outcome. Various surgical approaches, including orbitotomy, transcranial, and transnasal endoscopic techniques, should be considered based on foreign body location and extent. This case underscores the importance of early recognition, multimodal imaging, and timely surgical intervention in managing TOPI. Despite the high‐risk nature of such injuries, meticulous planning and prompt treatment can lead to excellent functional and neurological recovery.

**Summary**

- Transorbital penetrating intracranial injury (TOPI) from a wooden foreign body is rare and poses significant diagnostic and therapeutic challenges.
- Prompt imaging, early surgical intervention, and appropriate antibiotic therapy are crucial to prevent infections and complications.
- Timely intervention can lead to excellent recovery with preserved neurological and visual function.

## 1 Introduction

Transorbital penetrating intracranial injury (TOPI) is one of the rare cases of traumatic brain injury. The major cause of TOPI is missiles such as bullets in the war. Non‐missile foreign bodies such as iron rods, wood, nails, and needles are even more rare [1, 2, 3].

Penetrating Brain Injuries are associated with high mortality and morbidity due to damage to important structures such as major vessels and the brainstem, as well as the risk of infection. Vascular injury or injury to the brainstem might be fatal. The risk of infection is increased in the case of a wooden foreign body, although infection almost always occurs in delayed treatment. However, early treatment with surgical and nonsurgical approaches can reduce the chances of infection and hemorrhage; therefore, morbidity and mortality significantly [3].

Here we present a unique case of TOPI caused by a wooden foreign body. Despite the complexity of such an injury, our case demonstrated a successful clinical outcome with no long‐term morbidity or mortality. This case report was prepared in line with SCARE guidelines [4].

## 2 Case Presentation

### 2.1 History and Examination

A 58‐year‐old Nepali male with no prior ocular or systemic comorbidities presented to our emergency with chief complaint of fall from a 15‐ft tree 6 days back, during which he sustained direct trauma to his right eye, face, and head. He reported persistent pain and bleeding from the right eye and nose but denied loss of consciousness, vomiting, abnormal body movement, diplopia, or seepage of clear fluid from the eye. On examination, he was alert with stable vital signs and a Glasgow Coma Scale score of 15. Physical assessment revealed an incised wound over the right upper eyelid and a minor abrasion on the left leg, with no other external injuries. At presentation and during follow‐up, the patient underwent comprehensive neurological and ophthalmological evaluation, including visual acuity, extraocular movement, pupillary reflexes, fundoscopy, and confrontation visual fields. The patient did not have any signs of focal neurological deficits. Ophthalmologic evaluation showed significant chemosis and a medial canthus laceration in the right eye, a palpable firm mass within the right orbit, intact extraocular movements, clear bilateral corneas, and a normal fundus. Digital intraocular pressure assessments were within normal limits.

## 3 Methodology (Investigations, Differential Diagnosis and Management)

**FIGURE 1:**
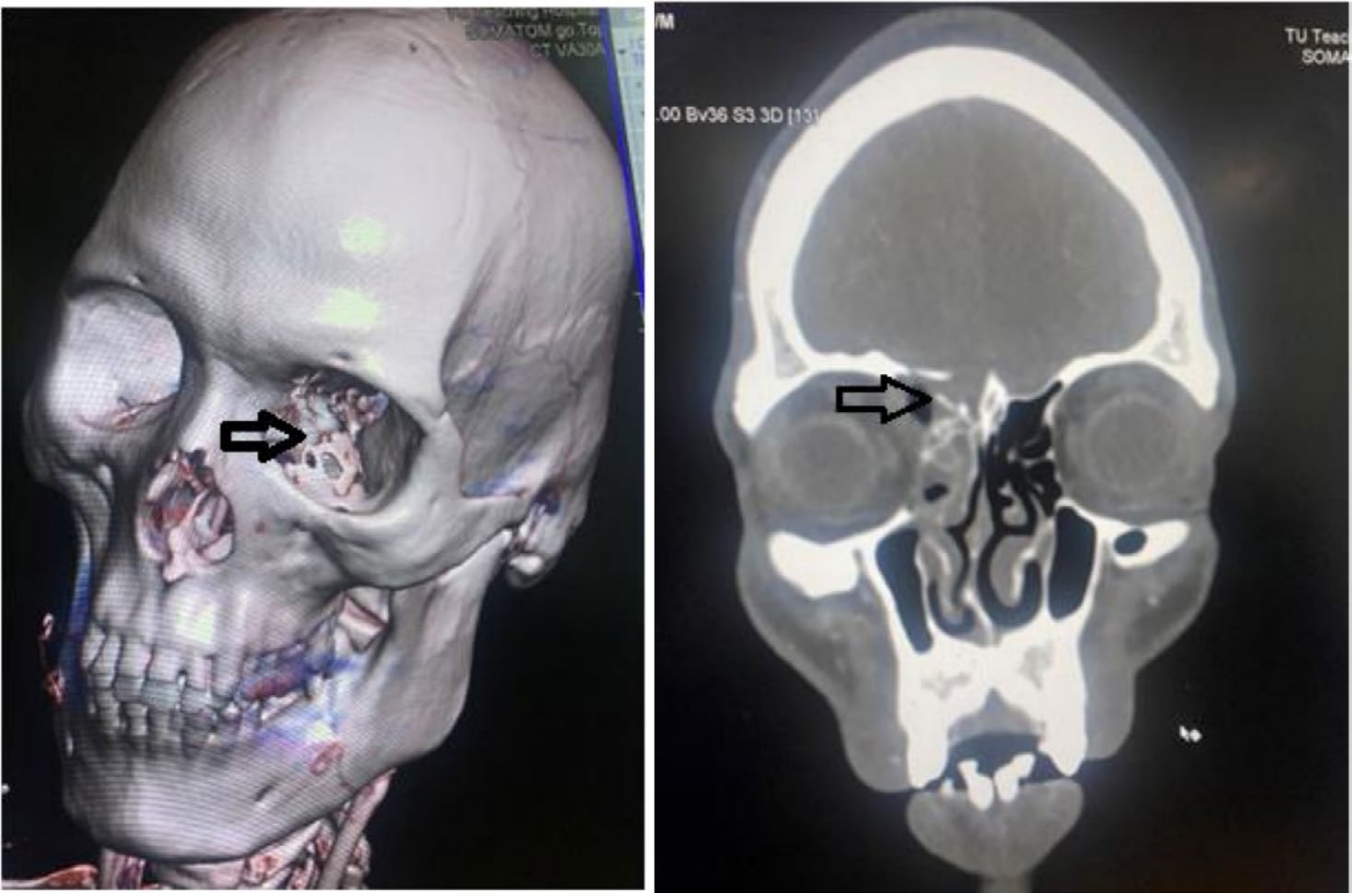
Comminuted fracture (arrow) of the superomedial orbital wall with fragments displaced medially into the right frontal lobe.

**FIGURE 2:**
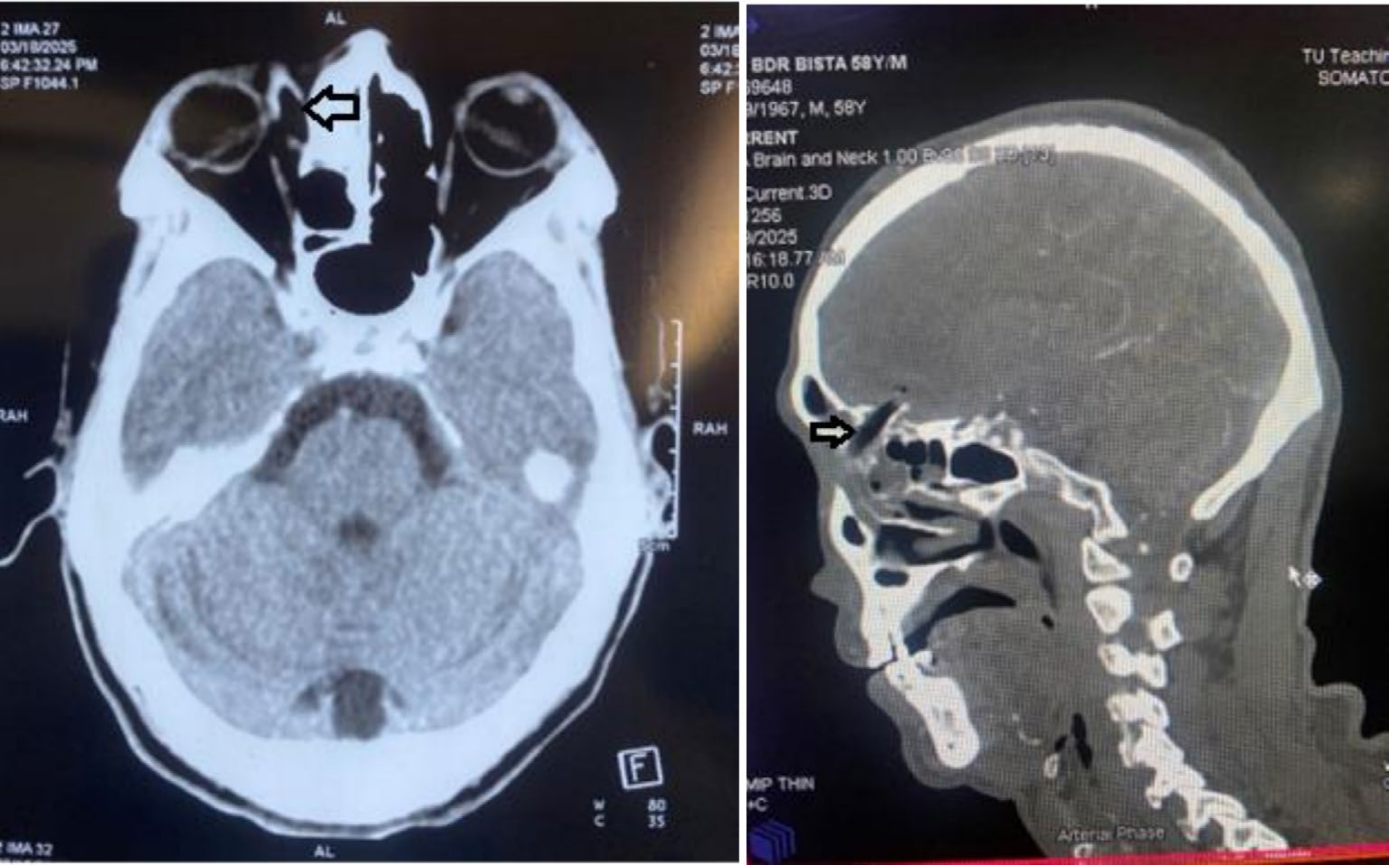
A parallel, heterogeneous track (arrow) in the medial extraconal space, suggestive of an intraorbital foreign body.

**FIGURE 3:**
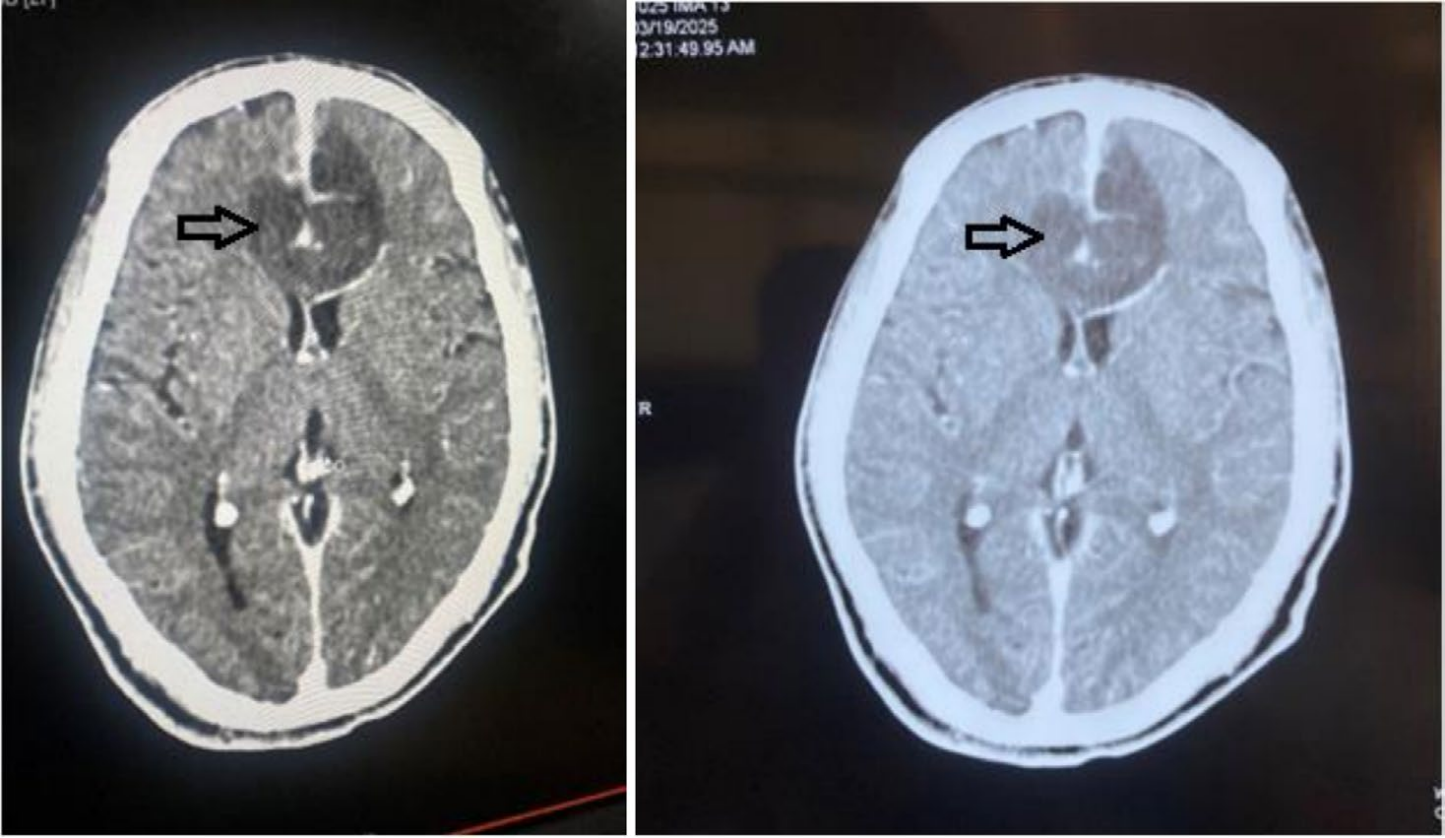
Hypodense area (arrow) in the bilateral frontal lobes.

Routine laboratory tests and an extended focused assessment with sonography for trauma (e‐FAST) were unremarkable. A non‐contrast computed tomography (CT) scan of the head and orbit revealed a comminuted fracture of the superomedial orbital wall with fragments displaced medially into the right frontal lobe (Figure 1). Additionally, a parallel, heterogeneous track measuring approximately 34.8 × 8.5 mm was identified in the medial extraconal space, suggestive of an intraorbital foreign body (Figure 2). The CT scan also revealed a conglomerated hypodense area in the left frontal lobe in the medial aspect adjacent to the interhemispheric falx and a hypodense area in the right frontal lobe in the medial aspect involving the genu of the corpus callosum suggesting edema or infarction in the ACA territory (Figure 3). A linear branching hyperdensity in the bilateral frontal lobes, suggestive of subarachnoid hemorrhage (SAH), was also noted in the CT scan (Figure 3). With this history, examination, and investigation, a diagnosis of a right orbital foreign body was made. Other differentials that were taken into consideration were orbital roof fracture with intracranial extension, intracranial vascular injuries, traumatic orbital hematoma, and intracranial contusion due to a penetrating injury.

**FIGURE 4:**
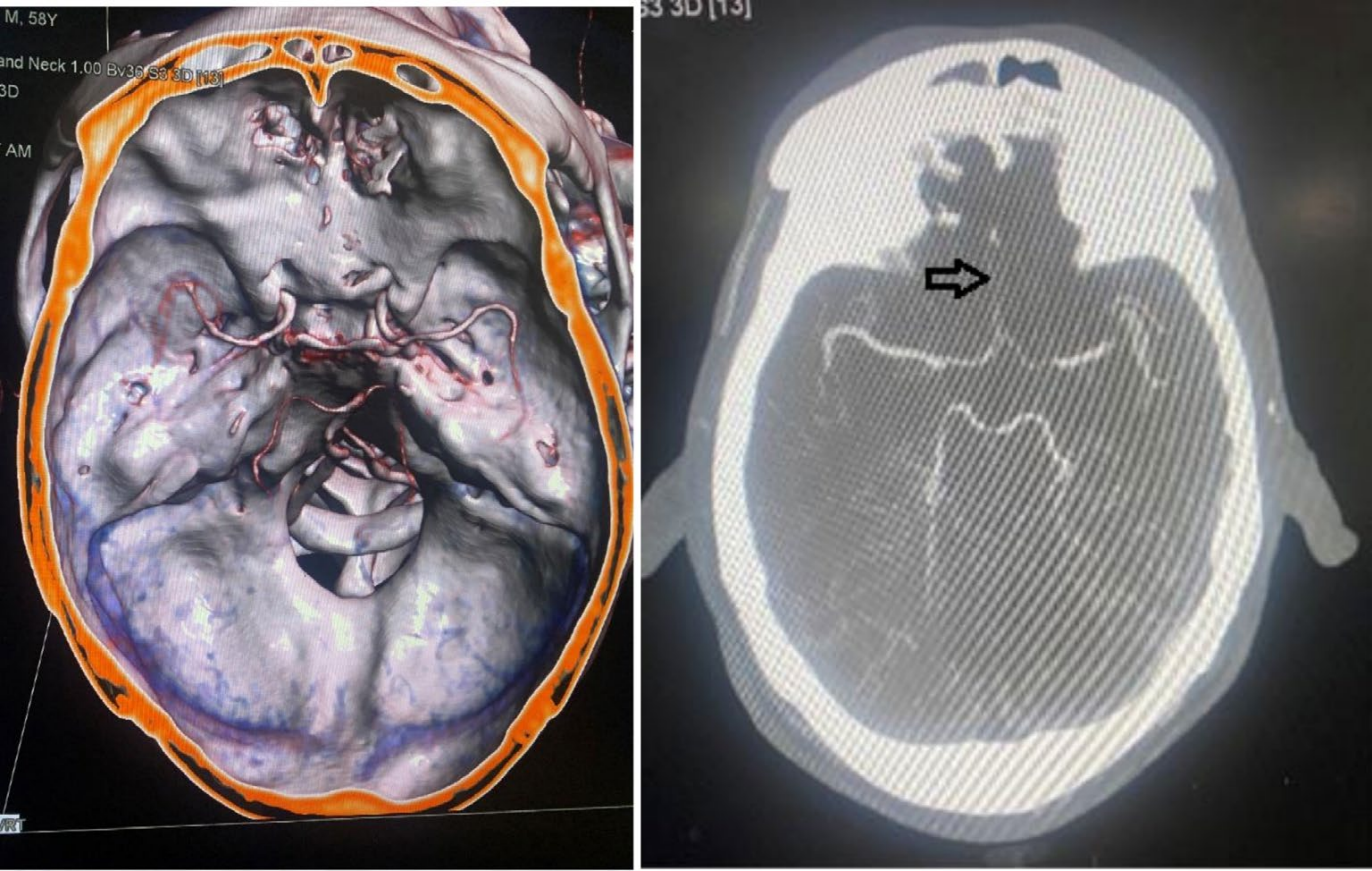
CT‐angiogram showing non‐opacification of A2 and attenuated caliber of A1 segment of anterior cerebral artery (ACA).

**FIGURE 5:**
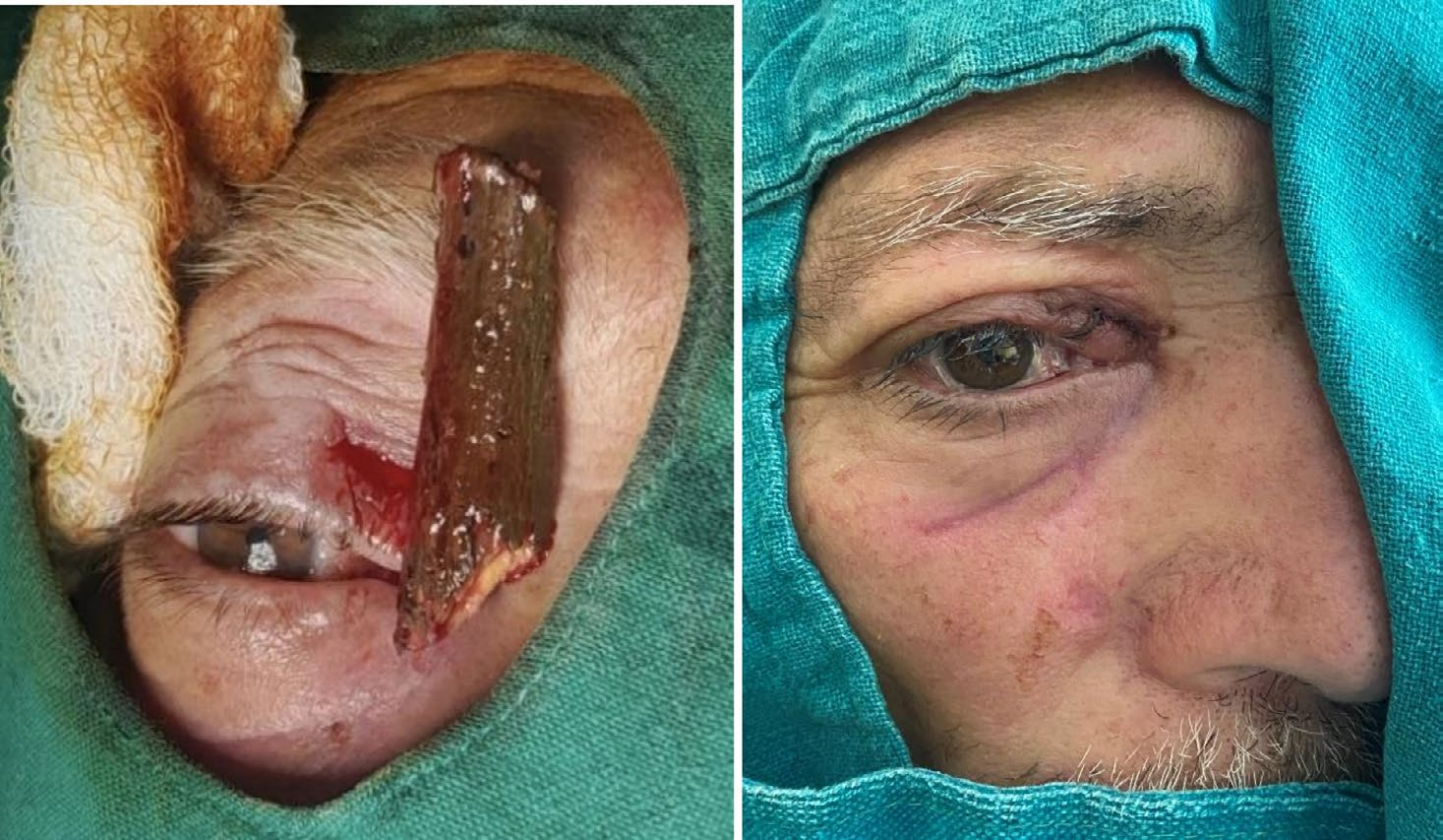
Intraoperative and postoperative image respectively.

The patient was promptly initiated on intravenous broad‐spectrum antibiotics to prevent infection. A neurosurgical consultation was obtained due to the intracranial extension of the fracture fragments. A CT angiogram of the head was done, which showed non‐opacification of A2 and attenuated caliber of the A1 segment of the anterior cerebral artery (ACA) suggesting impaired flow of blood in bilateral ACA (Figure 4). So, after discussion of the Neurosurgery and Ophthalmology teams, the transorbital approach was chosen over the transcranial approach for removal of the foreign body. Subsequently, he underwent a right medial orbitotomy under general anesthesia for exploration and removal of the wooden piece which measured about 6 × 2 cm. Intraoperatively, accessible loose bone fragments within the orbit were removed, while smaller fragments with stable positioning and no dural breach were left in situ. The decision aimed to minimize additional trauma to adjacent neurovascular structures. Intraoperative findings corroborated the imaging studies, and the procedure proceeded without complications. A sample for culture was sent from the site of the foreign body, but it did not reveal growth of any pathogen. The postoperative course was uneventful with no CSF leak and unimpaired vision (Figure 5). He was discharged after the completion of an antibiotic dose of 14 days. There were no issues at follow‐up with good wound healing and significant clinical improvement.

## 4 Discussion

Penetrating injuries of the eye and orbit are not uncommon nowadays. The most common cause of penetrating brain injuries is gunshots, either self‐inflicted or related to armed conflicts and warfare [5]. Iron rods, nails, or wood are other foreign bodies causing penetrating brain injuries. Transorbital penetrating intracranial injury by a wooden foreign body is extremely rare [3].

The structure of the orbit influences the risk of orbital injuries with intracranial penetration. Its horizontal pyramid shape directs penetrating objects toward the apex, where the superior orbital fissure and optic foramen provide a path to the brain. The superior orbital plate of the frontal bone is particularly vulnerable, especially in children, due to its thinness and lack of protective ridges. Reflex head movements during injury may further expose this weak point. The globe of the eye is well protected by strong scleral covering, periorbital fat, and the blink reflex, which helps shield it from direct penetration [6].

Detailed history and examination, including full neurological and ophthalmological examinations, is essential in the diagnosis and appropriate treatment of any patient diagnosed with transorbital intracranial trauma. As wooden foreign bodies are even rarer, they may present more diagnostic and therapeutic challenges. The air‐filled porous structure of dry wood can mimic pneumocephalus on a CT scan of the head, making the radiological diagnosis of an intracranial wooden foreign body injury challenging. The key differentiating feature of a wooden foreign body from pneumocephalus is the typical shape of the foreign body and different attenuation values. Dry wood typically has an attenuation range of −100 to −170 HU, while pneumocephalus measures between −600 and −1000 HU. However, CT attenuation values vary with time, as the water content of the wood can change with time, and so freshly cut wood that has a relatively high water content may mimic soft tissues in CT images [3]. Given these diagnostic challenges, detailed history and examination are very important because a CT scan alone poses difficulty in the diagnosis of TOPI of a wooden foreign body. MRI is a safe modality (as long as no metallic foreign body is suspected) with higher sensitivity compared to CT due to its ability to detect a wooden foreign body and soft tissue. Although, in cases of nonmetallic foreign bodies like wooden objects in our case, an MRI scan is the investigation of choice to determine the course of the foreign body and any associated intracranial damage. A CT scan of the brain and orbit was ordered, keeping in mind the economic factors, time‐consuming MRI procedure requiring patient cooperation, and readily available CT scans. A CT angiography or MR angiography is necessary when there is suspicion of a possible vascular injury. In such cases, angiography should also be performed to rule out traumatic aneurysm, which can develop soon after a penetrating injury. Therefore, the combination of multiple modalities of imaging is essential for correct diagnosis and surgical planning. In our case, we did a CT scan of the head followed by a CT angiogram, which showed evidence of fracture of the superomedial orbital wall with intracranial extension and impairment of blood flow in the bilateral ACA. Interestingly, the patient did not have any neurological deficits, suggesting that the vascular compromise would have occurred in a non‐eloquent area of the ACA territory.

Studies recommend early use of broad‐spectrum antibiotics covering common pathogens, including anaerobic and fungal coverage, especially in organic foreign bodies [7, 8]. In our case, the patient presented 6 days post‐injury. This delay increased the theoretical risk of infection and vascular compromise, but fortunately, he remained clinically stable with no neurological deterioration. Surgical planning was adapted accordingly, prioritizing infection control with early initiation of broad‐spectrum antibiotics. Surgical treatment should ideally be performed within 12 h of the injury to minimize the risk of infection‐related complications [9]. Reports indicate that vascular complications after penetrating brain injury vary widely, ranging from less than 5% to 40%. Angiography plays a vital role in diagnosing vascular complications due to the poor prognosis associated when they are not aggressively treated. The common vascular complications after penetrating brain injury include traumatic intracranial aneurysms or arteriovenous fistulas (AVFs), SAH, and vasospasm [10].

Intraorbital foreign bodies can be removed by orbitotomy procedures, transcranial approach, or transnasal endoscopic approach depending upon the extent of involvement by the foreign body and expertise of the surgeon. Transnasal endoscopic approach is adopted for a subset of foreign bodies which are placed more medially in the orbit and has been emphasized nowadays because of minimal soft tissue injury and no external scars [11]. Foreign bodies of the anterior two‐thirds of the orbital cavity might be approached extra‐cranially by orbitotomy [12]. Foreign bodies impacted in the medial aspect of the orbit, medial to the optic nerve, are accessed via a medial orbitotomy [13]. Transcranial approach is adopted when the foreign bodies are not readily removable because of their location such as retrobulbar or apical area, when they have considerable intracranial extension, or when there is need of better control to the neurovascular structures during the surgery. However, this approach needs technical competence and is time‐consuming [12, 14]. Furthermore, foreign bodies extending beyond the superior orbital fissure or complex cases in which they are deeply embedded or located near critical structures such as the optic nerve, carotid artery, or the skull base may need a combination of the above approaches [12, 15]. In the presence of the neurosurgery team, the ophthalmologist performed medial orbitotomy as the wooden body was on the anterior two thirds of the orbital cavity.

Infectious complications are not uncommon after penetrating brain injury, and they are also associated with higher morbidity and mortality rates [9]. Infectious complications are more frequent when cerebrospinal fluid leaks, air sinus wounds, trans‐ventricular injuries, or those crossing the midline occur. *Staphylococcus aureus* is the most frequently associated organism. However, gram‐negative bacteria also frequently cause intracranial infection after penetrating brain injury. Based on available literature, it is recommended that broad‐spectrum antibiotics should be instituted in all PBI cases and must be started as soon as possible [9, 16]. Antibiotics should be changed after the culture sensitivity report. Similarly, vascular complications such as pseudoaneurysm or AV fistula can develop days to weeks post‐injury. In our case, no follow‐up CTA/MRA was performed due to resource constraints, although the patient remained asymptomatic at follow‐up. This was the limitation of our case report.

## 5 Conclusion

We present a unique case of TOPI from a wooden foreign body. Penetrating orbital injury by wooden fragments that extend into the intracranial space is rare but potentially sight‐threatening and sometimes life‐threatening. Wooden fragments are notoriously difficult to detect with CT and need appropriate clinical correlation. CT angiography helps in proper delineation of possible vascular injury. Early surgical evacuation and treatment with appropriate antibiotics are essential to prevent infection and can result in excellent recovery of ocular function and preservation of neurovascular integrity.

## Author Contributions

**Uttam Chaulagain:** conceptualization, investigation, methodology, project administration, resources, writing – original draft, writing – review and editing. **Kapil Khanal:** conceptualization, methodology, resources, writing – original draft, writing – review and editing. **Kailash Mani Pokhrel:** resources, visualization, writing – original draft. **Nischal Soti:** conceptualization, investigation, methodology, resources, supervision, visualization. **Urusha Naaz:** conceptualization, investigation, methodology, project administration, resources, supervision, visualization. **Prabhat Jha:** conceptualization, investigation, methodology, supervision. **Dipendra K. Shrestha:** conceptualization, investigation, methodology, supervision. **Sushil Kumar Shilpakar:** conceptualization, investigation, methodology, supervision, validation.

## Consent

Written informed consent was obtained from the patient for the publication of this case report and accompanying images. A copy of written consent is available for review by the editor in chief of this journal on request.

## Conflicts of Interest

The authors declare no conflicts of interest.

## Data Availability

The data used in this article are available upon request from the authors.
